# Comparación de los perfiles de resistencia antimicrobiana de *Escherichia coli* uropatógena e incidencia de la producción de betalactamasas de espectro extendido en tres establecimientos privados de salud de Perú

**DOI:** 10.7705/biomedica.4772

**Published:** 2020-08-20

**Authors:** Pool Marcos-Carbajal, Marco Galarza-Pérez, Salomón Huancahuire-Vega, Miguel Otiniano-Trujillo, Javier Soto-Pastrana

**Affiliations:** 1 Laboratorio de Microbiología, Escuela Profesional de Medicina Humana, Universidad Peruana Unión, Lima, Perú Universidad Peruana Unión Universidad Peruana Unión Lima Peru; 2 Laboratorio de Referencia Nacional de Biotecnología y Biología Molecular, Instituto Nacional de Salud, Lima, Perú Instituto Nacional de Salud Lima Perú; 3 Laboratorio de Microbiología, Hospital Nacional Docente “Madre Niño San Bartolomé”, Lima, Perú Hospital Nacional Docente “Madre Niño San Bartolomé” Lima Perú

**Keywords:** Enterobacteriaceae, pruebas antimicrobianas de difusión por disco, infecciones urinarias, beta-lactamasas, resistencia a medicamentos, Perú, Enterobacteriaceae, disk diffusion antimicrobial tests, urinary tract infections, beta-lactamases, drug resistance, Perú

## Abstract

**Introducción.:**

La aparición de enterobacterias multirresistentes y productoras de betalactamasas de espectro extendido en pacientes ambulatorios con infecciones urinarias representa un problema de salud pública en Perú.

**Objetivo.:**

Comparar los perfiles de resistencia de *Escherichia coli* uropatógenas e identificar los fenotipos de cepas productoras de betalactamasas de espectro extendido en tres establecimientos privados de salud localizados en las regiones de la costa, la sierra y la selva de Perú.

**Materiales y métodos.:**

Se llevó a cabo durante el 2016 un estudio descriptivo de 98 muestras de orina de pacientes con infección urinaria, 35 procedentes de Lima (costa), 38 de Juliaca (sierra) y 25 de Iquitos (selva), en el que se determinó la sensibilidad antimicrobiana utilizando ocho discos antibióticos.

Asimismo, se evaluó la producción de betalactamasas de espectro extendido con discos de cefotaxima, de ceftazidima o de su combinación, con ácido clavulánico en agar Mueller-Hinton.

**Resultados.:**

Se identificaron 18 perfiles de resistencia que incluían desde los sensibles a todos los antibióticos hasta los resistentes simultáneamente a siete antibióticos, con el 18,4 % de aislamientos resistentes a un antibiótico y el 54,0 % de multirresistentes. Se detectó producción de betalactamasas en el 28,6 % de las cepas procedentes de la región de Puno. También, se observó un mayor número de casos en el rango de edad de 31 a 45 años con resistencia a ceftazidima, ceftriaxona, gentamicina y trimetoprim-sulfametoxazol en el establecimiento de salud de Puno.

**Conclusión.:**

Los perfiles de resistencia variaron según la localización geográfica del establecimiento de salud, observándose mayor resistencia a los antibióticos en la región de la sierra de Perú, con el 28,6 % de cepas productoras de betalactamasas de espectro extendido.

## Abstract (en)

**Introduction:**

The appearance of multidrug-resistant and beta-lactamase producing enterobacteria in outpatient care facilities represent a public health problem in Perú.

**Objective:**

To compare the resistance profiles of uropathogenic *Escherichia coli* and to identify extended-spectrum beta-lactamase-producing phenotypes in three private health facilities located in the Peruvian coast, Andean and jungle regions.

**Materials and methods:**

We conducted a descriptive study on 98 urine samples from Lima (coast), Juliaca (Andean region) and Iquitos (jungle region) during 2016. We determined the antimicrobial susceptibility in 35 samples from Lima, 38 from Juliaca and 25 from Iquitos using eight antibiotic disks in samples from patients diagnosed with urinary infection. We also evaluated the production of extended-spectrum beta-lactamases with cefotaxime and ceftazidime disks and a combination of both with clavulanic acid on Mueller-Hinton agar.

**Results:**

We identified 18 resistance profiles ranging from those sensitive to others simultaneously resistant to seven antibiotics: 18.4% resistant to one and 54.0% to multiple antibiotics. We detected beta-lactamase production in 28.6% of the strains from the Puno region. Likewise, we observed a greater number of cases with resistance to ceftazidime, ceftriaxone, gentamicin, and trimethoprim-sulfamethoxazole in Puno’s health facility in patients within the 31 to 45 year age range.

**Conclusion:**

Resistance profiles varied according to the geographical location of the health facilities under study. Resistance to antibiotics was higher in the Andean region with 28.6% of strains producing extended-spectrum beta-lactamases.

La incidencia de infecciones urinarias es una de las principales razones por las que los médicos prescriben tratamientos con antibióticos. Estas infecciones son causadas por enterobacterias, principalmente *Escherichia coli* y las del género *Klebsiella*^1^.

A nivel mundial, se registra una alta tasa de resistencia de las enterobacterias a los antibióticos en las infecciones urinarias. El interés clínico se concentra en un grupo de estas bacterias con actividad enzimática de betalactamasas de espectro extendido (BLEE) resistentes a las cefalosporinas de tercera generación ^2^. Dichas betalactamasas son muy eficientes en la regulación de los genes que confieren resistencia a los antibióticos.

La prevalencia de las cepas con BLEE varía según la ubicación geográfica y, en Latinoamérica, la prevalencia es mayor que en los Estados Unidos y en Europa ^3^. Se han reportado más de 200 variantes de BLEE en casi todas las especies de enterobacterias asociadas con los genes *CTX-M*, *SHV*, *TEM*, *PER* y *OXA 4*. Algunas de estas enzimas son específicas para hidrolizar penicilinas o cefalosporinas, en tanto que otras tienen un espectro amplio de actividad, incluida la inactivación de la mayoría de los antibióticos betalactámicos ^4^.

La familia de las enzimas CTX-M se subdivide en 124 variantes y constituye el grupo de BLEE más predominante. Esta familia de enzimas de *E. coli* es la que se ha diseminado más rápidamente a nivel mundial y está asociada con brotes hospitalarios e infecciones adquiridas en la comunidad ^5^. La mayoría de los microorganismos productores de BLEE constituye un problema cotidiano en las instituciones hospitalarias; sin embargo, en diversos estudios también se las ha encontrado en la comunidad, en sedimentos de ríos urbanos y en letrinas privadas y de uso común en algunas zonas ^6^^,^^7^. Factores de riesgo como el uso de catéter urinario, la hospitalización, las comorbilidades, una edad de más de 65 años y la presencia de enfermedades serias, también juegan un papel muy importante en la aparición de bacterias uropatógenas con capacidad hidrolítica por BLEE ^8^.

En estas condiciones de gran resistencia bacteriana, es necesario impulsar el uso racional y apropiado de los antibióticos, es decir, disminuir la automedicación y cumplir los tratamientos de manera responsable ^9^. En Perú, hay subestimación de la prevalencia de las bacterias productoras de BLEE debido a su difícil detección en el laboratorio y a la escasa implementación de metodologías moleculares para la identificación de los genes relacionados con la actividad hidrolítica de algunos antibióticos. Hasta ahora, los estudios se han enfocado en los departamentos de la costa, por lo que es necesario analizar los perfiles de resistencia de estos agentes patógenos en otros establecimientos de salud de las zonas andina y de selva ^10^.

En este contexto, el objetivo del estudio fue comparar los perfiles de resistencia antibiótica y la incidencia del fenotipo de las BLEE en cepas de *E. coli* aisladas de muestras de orina de pacientes ambulatorios procedentes de tres establecimientos privados de salud localizados en diferentes departamentos de Perú.

## Materiales y métodos

### Pacientes y material biológico

Durante el 2016, se llevó a cabo un estudio descriptivo en pacientes de 1 a 91 años de edad con infección urinaria, atendidos de manera ambulatoria en tres establecimientos privados de salud : en Lima, la Clínica Good Hope; en Puno, la Clínica Americana Juliaca, y en Iquitos, la Clínica Ana Stahl. Se analizaron 98 muestras de orina con aislamiento microbiológico de *E. coli*.

### Determinación de la resistencia antibiótica mediante métodos microbiológicos

Los aislamientos de *E. coli* obtenidos de los urocultivos se enviaron al Laboratorio de Microbiología de la Escuela de Medicina de la Universidad Peruana Unión para confirmar, mediante pruebas bioquímicas convencionales y el sistema automatizado Vitek 2™ (bioMérieux, Inc., Hazelwood, MO, USA), la sensibilidad a ceftazidima (CAZ), ceftriaxona (CRO), ciprofloxacina (CIP), gentamicina (GEN), trimetoprim-sulfametoxazol (SXT), imipenem (IPM), amikacina (AK) y nitrofurantoína (NIT). La presencia de BLEE se detectó en placas de agar Mueller-Hinton y se emplearon discos de cefotaxima (CTX), cefotaxima-ácido clavulánico (CTX/CXT-CLA), ceftazidima (CAZ) y ceftazidima-ácido clavulánico (CAZ/CAZ-CLA).

Para el control de calidad de las pruebas de sensibilidad, se usaron las cepas *Staphylococcus aureus* ATCC 25923, *E. coli* ATCC 25922, *Pseudomonas aeruginosa* ATCC 27853 y *Klebsiella pneumoniae* ATCC 700603 como controles positivos para la producción de BLEE, y *E. coli* ATCC 25922 como control negativo. Todos los procedimientos se ajustaron a los estándares del *Clinical and Laboratory Standards Institute* (CLSI) ^11^.

### Análisis estadístico

Los datos obtenidos de las variables microbiológicas y clínicas se procesaron en Microsoft Excel 2013, y el cálculo de la proporción de cepas sensibles y resistentes a antibióticos, se ajustó a las normas del CLSI.

### Consideraciones éticas

Los tres establecimientos de salud aprobaron el protocolo de estudio. Se garantizó la confidencialidad de los datos de los pacientes. El estudio fue aprobado por el Comité de Investigación y Ética de la Universidad Peruana Unión.

## Resultados

### Características de los pacientes ambulatorios

De los 98 pacientes atendidos ambulatoriamente, el 26,5 % entre los 31 y los 45 años de edad, y el 24,5 % mayores de 61 años, tuvieron cultivos positivos. En cuanto al establecimiento de salud, el 38,8 % de los cultivos positivos procedía de Puno, el 35,7 % de Lima y el 25,5 % de Iquitos.

### Perfiles de resistencia antibiótica en aislamientos de Escherichia coli

Se identificaron microbiológicamente 18 perfiles diferentes de resistencia antimicrobiana (cuadro 1). El fenotipo ‘pansensible’ (perfil XVIII) fue el más frecuente (27,6 %) en los aislamientos, seguido del perfil XVII de resistencia a un antibiótico (18,4 %), del perfil XVI sensible a dos antibióticos (16,3 %), de la combinación de los perfiles IV, V, VI, VII, VIII y IX resistente a cinco antibióticos (15,3 %), de los perfiles X, XI y XII resistentes a cuatro antibióticos (9,2 %), de los perfiles XIII, XIV y XV resistentes a tres antibióticos (7,1 %), y de los perfiles II y III resistentes a seis antibióticos (5,1 %). Solamente se presentó un caso con el perfil I de resistencia a siete antibióticos. Entre los perfiles con resistencia a cinco antibióticos, el perfil IV, resistente a ceftazidima, ceftriaxona, ciprofloxacino, gentamicina y trimetoprim-sulfametoxazol, fue el más frecuente (9,2 %).

**Cuadro 1:** Perfiles de resistencia en 98 aislamientos de *E. coli* procedentes de tres establecimientos de salud en Perú

Perfil	Perfiles de resistencia antibiótica	n/N	(%)
I	CAZ-CRO-CIP-GEN-SXT-IPM-AK	1/98	(1,0)
II	CAZ-CRO-CIP-GEN-SXT-IPM	4/98	(4,1)
III	CAZ-CRO-CIP-GEN-SXT-NIT	1/98	(1,0)
IV	CAZ-CRO-CIP-GEN-SXT	9/98	(9,2)
V	CAZ-CRO-CIP-GEN-IPM	1/98	(1,0)
VI	CAZ-CRO-CIP-GEN-NIT	2/98	(2,0)
VII	CAZ-CRO-CIP-SXT-AK	1/98	(1,0)
VIII	CAZ-CRO-GEN-SXT-IPM	1/98	(1,0)
IX	CAZ-GEN-SXT-NIT-AK	1/98	(1,0)
X	CAZ-CRO-CIP-GEN	1/98	(1,0)
XI	CAZ-CRO-CIP-SXT	7/98	(7,1)
XII	CIP-GEN-SXT-NIT	1/98	(1,0)
XIII	CIP-GEN-SXT	4/98	(4,1)
XIV	CIP-SXT-IPM	1/98	(1,0)
XV	GEN-SXT-NIT	2/98	(2,0)
XVI	Resistencia a dos antibióticos	16/98	(16,3)
XVII	Resistencia a un antibiótico	18/98	(18,4)
XVIII	Pansensible	27/98	(27,6)
	Total	98	(100)

CAZ: ceftazidima; CRO: ceftriaxona; CIP: ciprofloxacino; GEN: gentamicina;

### Comparación de la resistencia antibiótica por establecimiento de salud

Con respecto al número de casos, se observó que la resistencia contra trimetoprim-sulfametoxazol fue más frecuente, 61,2 % de los casos, en los tres establecimientos de salud. El establecimiento de salud de Puno presentó el 26,5 % de los casos, seguido del de Lima con el 18,4 % y el de Iquitos con el 16,3 %.

La ciprofloxacina fue el segundo antibiótico con más casos de resistencia en los tres establecimientos (48,0 %). Los establecimientos de salud de Puno y Lima presentaron 18,4 % casos cada uno, en tanto que el 11,2 % de los casos se presentó en Iquitos.

La resistencia a ceftazidima se encontró en 31,6 % de los casos y en Puno se presentó el 15,3 % de ellos, seguido de Lima con 9,2 %, e Iquitos con 7,1 %.

En cuanto a la ceftriaxona, la resistencia se presentó en 30,6 % de los casos: en Puno, se presentó el 14,3 % de ellos, seguido de Lima con el 9,2 %, e Iquitos con el 7,1 %.

La resistencia a la gentamicina se dio en el 31,6 % de los casos. En Puno, se presentó el 13,3 % de ellos, seguido de Lima e Iquitos con 92 % de casos en cada establecimiento.

**Figura 1:**
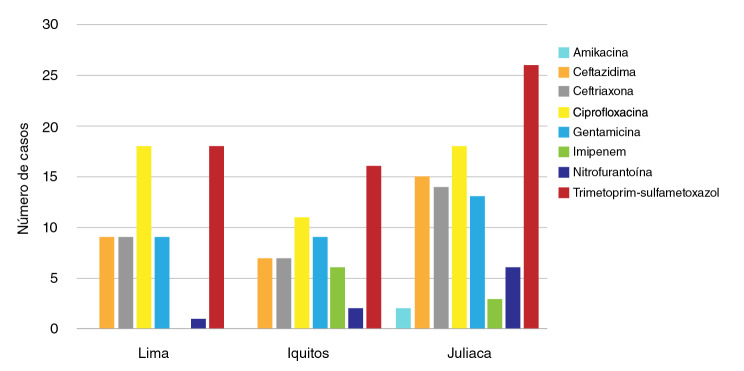
Número de casos de resistencia antibiótica según establecimiento de salud. Cada barra en color representa el número de casos resistentes por antibiótico. No se observó resistencia a la amikacina y el imipenem en Lima, ni a la amikacina en Iquitos.

Con los antibióticos imipenem, nitrofurantoína y amikacina, se presentó la resistencia más baja en las cepas analizadas. La resistencia a imipenem estuvo presente en el 9,2 % de los casos y, en Iquitos y Puno, se presentaron 6,1 y 3,1 % de los casos, respectivamente. No se encontraron casos de resistencia en Lima. La resistencia a nitrofurantoína se registró en el 9,2 % de los casos. En Puno se presentó el 6,1 % de ellos, seguido de Iquitos con el 2,0 % y Lima con el 1,0 %. La resistencia a amikacina se encontró en el 2,0 % de los casos, todos procedentes del establecimiento de salud de Puno (figura 1).

### Perfiles de resistencia con el fenotipo de las BLEE

En 28 (28,6 %) cepas de *E. coli* con el fenotipo de las BLEE, se identificaron 10 perfiles de resistencia (cuadro 2). El perfil IV, resistencia a cinco antibióticos (ceftazidima, ceftriaxona, ciprofloxacino, gentamicina y trimetoprim-sulfametoxazol), se identificó en el 32,1 % de las cepas. El perfil XI a cuatro antibióticos (ceftazidima, ceftriaxona, ciprofloxacino y trimetoprim- sulfametoxazol) se identificó en el 25,0 % de las cepas. Se identificaron seis perfiles resistentes (III, V, VII, IX, X y XVI), tres de los cuales fueron resistentes a seis y cinco antibióticos, y otros tres perfiles presentaron resistencia a cuatro y dos antibióticos.

**Cuadro 2:** Perfil de resistencia en 28 cepas de *E. coli* con fenotipo productor de BLEE, procedentes de tres establecimientos de salud en Perú

Perfil	Número de antibióticos	Perfiles de resistencia antibiótica	Número de cepas	%
II	6	CAZ-CRO-CIP-GEN-SXT-IPM	4	14,3
III	6	CAZ-CRO-CIP-GEN-SXT-NIT	1	3,6
IV	5	CAZ-CRO-CIP-GEN-SXT	9	32,1
V	5	CAZ-CRO-CIP-GEN-IPM	1	3,6
VI	5	CAZ-CRO-CIP-GEN-NIT	2	7,2
VII	5	CAZ-CRO-CIP-SXT-AK	1	3,6
IX	5	CAZ-GEN-SXT-NIT-AK	1	3,6
X	4	CAZ-CRO-CIP-GEN	1	3,6
XI	4	CAZ-CRO-CIP-SXT	7	25,0
XVI	2	CAZ-CRO	1	3,6
		Total	28	100

CAZ: ceftazidima; CRO: ceftriaxona; CIP: ciprofloxacino; GEN: gentamicina; SXT: trimetoprim-sulfametoxazol; IPM: imipenem; AK: amikacina; NIT: nitrofurantoína

**Figura 2:**
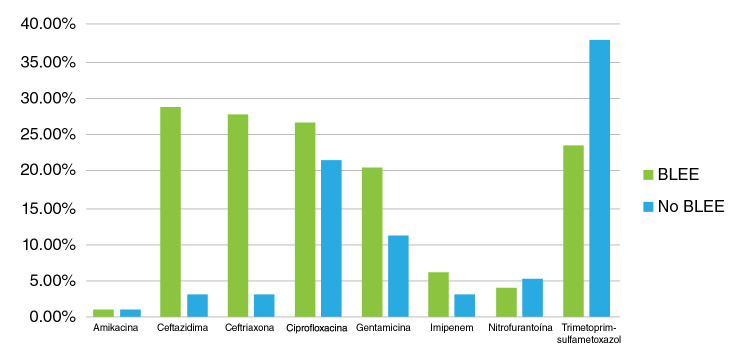
Representación porcentual de casos con el fenotipo productor de BLEE y el no productor. Las barras verdes indican la resistencia por antibiótico en cepas con BLEE y las azules, la resistencia por antibiótico en cepas sin BLEE.

Por otro lado, se observó una acentuada diferencia entre los perfiles de resistencia antibiótica de las cepas de *E. coli* productoras de BLEE y aquellas no productoras (figura 2). El fenotipo de las BLEE registró un mayor porcentaje de resistencia a los antibióticos ceftazidima, ceftriaxona, ciprofloxacina y gentamicina, en comparación con los aislamientos sin ese fenotipo. Los porcentajes de resistencia a los antibióticos mencionados fueron de 28,6, 27,6, 26,5 y 20,4 % en las cepas con el fenotipo de las BLEE, en tanto que en los otros fenotipos fueron de 3,1, 3,1, 21,4 y 11,2 %, respectivamente.

Los resultados se invirtieron con los antibióticos nitrofurantoína y trimetoprim-sulfametoxazol. Los fenotipos de las BLEE registraron porcentajes de resistencia de 4,1 y 23,5 %, respectivamente, en tanto que en los otros, los porcentajes de resistencia fueron de 5,1 y 37,8 %, respectivamente.

## Discusión

En este estudio se presenta un reporte preliminar de la alta tasa de multirresistencia en establecimientos de salud ubicados en las zonas de la costa, la sierra y la selva de Perú. Se confirmó la existencia de cepas de *E. coli* productoras de BLEE en los tres establecimientos de salud estudiados, específicamente en pacientes ambulatorios. La tasa encontrada (28,6 %) es menor que las de algunos estudios previos, en los que solo se consideró la zona de la costa (principalmente Lima) ^12^.

MacGowan, *et al.*, evaluaron los patrones de sensibilidad de las enterobacterias en 23 centros de 10 países latinoamericanos, entre ellos, Perú. Sus datos confirmaron la creciente frecuencia de microorganismos con el fenotipo productor de BLEE: 31,4 % de ellos en infecciones adquiridas en la comunidad y 24,9 % en las hospitalarias ^13^.

Algunos estudios de la zona costera de Perú han evidenciado la gran prevalencia de bacterias productoras de betalactamasas de espectro extendido. Arce, *et al.*, analizaron 66 cepas de *E. coli* con el fenotipo de las BLEE en pacientes hospitalizados, y confirmaron que el 60,6 % de ellas presentaba el gen *blaTEM*, el 12,2 % el gen *blaSHV* y el 27,2 % no presentaba ninguno de los dos genes estudiados ^14^. Rivera, *et al.*, por su parte, determinaron el genotipo de 15 cepas de enterobacterias productoras de betalactamasas de tipo *TEM* y *CTX-M*, aisladas de superficies de ambientes hospitalarios. En 11 cepas se identificaron ambos genes, tres presentaron solamente el *CTX-M* y una no presentó ninguno de los genes analizados ^15^. Arce, *et al.*, analizaron 35 cepas de *E. coli*, de las cuales el 51,4 % presentó el fenotipo de las BLEE y el genotipo *CTX-M* en urocultivos de pacientes mayoritariamente ambulatorios ^16^. Yábar, *et al.*, determinaron la incidencia de cepas productoras de BLEE en 353 muestras provenientes de diversos servicios en hospitales, incluidos los de urgencias.

En la población pediátrica, la incidencia de BLEE fue del 16,3 % en tanto que en la población adulta fue de 31,1 %. Además, el 63,6 % de las muestras provenía de pacientes ambulatorios. La presencia del fenotipo de las BLEE se asoció con la hospitalización en pediatría y con el uso de pañal, y con vejiga neurogénica en adultos ^17^. Más recientemente, Grandez, *et al.,* analizaron la frecuencia del fenotipo de las BLEE en *E. coli* durante tres años consecutivos y encontraron valores de 37,5, 47,0 y 50,1 %, respectivamente, en muestras de pacientes hospitalizados en un hospital de referencia en Lima ^18^.

En cuanto a la resistencia a los antibióticos, con los aminoglicósidos se encontraron bajas tasas de resistencia (menores de 15 %) en los tres establecimientos de salud estudiados. La resistencia a amikacina solo se registró en el 2,0 % en el establecimiento de salud de Puno. Considerando estos resultados, la gentamicina y la amikacina podrían usarse como tratamiento de primera línea en los pacientes con infección urinaria por cepas de *E. coli* multirresistentes. La nitrofurantoína, indicada en el tratamiento de primera línea en casos de infecciones urinarias ^19^, fue otro de los antibióticos con baja tasa de resistencia (menor de 10 %) en los tres establecimientos de salud. Por otro lado, en la actualidad el uso de fosfomicina como tratamiento para cepas con el fenotipo de las BLEE puede ser una alternativa terapéutica frente a cepas multirresistentes, ya que la sensibilidad puede ser de más del 70 % ^20^.

Una de las limitaciones del estudio fue el bajo número de muestras de orina de cada establecimiento de salud; sin embargo, los resultados son importantes al evidenciar la diferencia de perfiles de resistencia a los antibióticos según la zona geográfica y la disponibilidad de los medicamentos más adecuados para el tratamiento de las infecciones urinarias.

La importancia del estudio de los perfiles de resistencia antibiótica en diferentes áreas geográficas es que es una herramienta útil para vigilar el comportamiento de las bacterias en brotes locales. Asimismo, brinda información clínica para el tratamiento empírico de la infección urinaria ^13^.

En conclusión, los resultados preliminares de este estudio en establecimientos privados de salud en tres diferentes zonas geográficas de Perú deben considerarse como una alerta nacional sobre la necesidad de determinar la tasa de incidencia real en todo el país, implementar las acciones necesarias para controlar la circulación de cepas multirresistentes y llevar a cabo nuevos estudios sobre los factores de riesgo en pacientes ambulatorios, con el fin de desarrollar políticas de prevención.
